# The efficacy of a brief intervention to reduce alcohol misuse in patients with HIV in South Africa: study protocol for a randomized controlled trial

**DOI:** 10.1186/1745-6215-13-190

**Published:** 2012-10-09

**Authors:** Diana Huis in ’t Veld, Linda Skaal, Karl Peltzer, Robert Colebunders, John V Ndimande, Supa Pengpid

**Affiliations:** 1 Department of Epidemiology and Social Medicine, University of Antwerp, Universiteitsplein 1, Antwerp (Wilrijk), 2610, Belgium; 2 FWO Research Foundation Flanders, Egmontstraat 5, Brussels, 1000, Belgium; 3 Department of Clinical Sciences, Institute of Tropical Medicine, Nationalestraat 155, Antwerp, 2000, Belgium; 4 Department of Social and Behavioral Health Sciences, University of Limpopo, PO Box 215, Medunsa, 0204, Pretoria, South Africa; 5 Department of Health System Management and Policy, University of Limpopo, PO Box 215, Medunsa, 0204, Pretoria, South Africa; 6 HIV/AIDS, STIs & TB (HAST) Research Programme, Human Sciences Research Council, 134 Pretorius Street, Pretoria, 0002, South Africa; 7 Department of Psychology, University of Limpopo, Turfloop Campus, Sovenga, 0727, South Africa; 8 Department of Family Medicine and Primary Health Care, University of Limpopo, P.O. Box 222, Medunsa, 0204, Pretoria, South Africa

**Keywords:** Alcohol, HIV, South Africa, Intervention

## Abstract

**Background:**

Alcohol abuse comes with risks for increased morbidity and mortality among patients with HIV. This study aims to determine the prevalence of alcohol use and other risk factors in a sample of primary care patients with HIV in South Africa and to assess a brief intervention to reduce the use of alcohol in this group.

**Methods/Design:**

A single-blinded randomized controlled trial is designed to determine the efficacy of a brief intervention to reduce hazardous alcohol use in patients with HIV. The study will be carried out on out-patients with HIV in two primary healthcare HIV clinics near Pretoria, South Africa. Alcohol use will be assessed with the Alcohol Use Disorder Identification Test questionnaire. Other data that will be collected relate to health-related quality of life, depression, sexual behavior, internalized AIDS stigma, HIV-related information and adherence to antiretroviral therapy (self-reported 7-day recall of missed doses, Visual Analog Scale and pill count). The intervention consists of a brief counseling session to reduce alcohol risk; the control group receives a health education leaflet.

**Discussion:**

The findings will be important in the public health setting. If the intervention proves to be efficient, it could potentially be incorporated into the HIV care policy of the Ministry of Health.

**Trial registration:**

Pan African Clinical trial Registry: PACTR201202000355384

## Background

The use of alcohol in South Africa is among the highest in Africa [1] with a total adult per-capita consumption of 9.5 l pure alcohol per year, and a consumption of 34.91 l pure alcohol per year (men 39.64 l, women 23.84 l) among people that drink alcohol [2]. Since South Africa also has one of the highest prevalences of HIV in the world (prevalence estimated to be 17.8% among adults aged 15 to 49 in 2009) [3], it is possible that a considerable number of persons living with HIV engage in hazardous or harmful alcohol use. Hazardous drinking is defined as a quantity or pattern of alcohol consumption that places patients at risk for adverse health events, while harmful drinking is defined as alcohol consumption that results in adverse events (for example, physical or psychological harm) [4]. Prevalence data on (hazardous) alcohol use in patients with HIV in Africa vary since different measuring tools were used in different study populations. A systematic review by Nakimuli-Mpungu and colleagues on alcohol use, depression and adherence to antiretroviral therapy (ART) in patients with HIV showed a prevalence of alcohol use between 2.5 and 51%. The prevalence of alcohol use disorders ranged from 2.5 to 31% [5]. Myer and colleagues reported a prevalence of alcohol dependence/abuse of 7% in patients with HIV in Cape Town [6].

Studies in several African countries have shown a causal link between alcohol use and HIV, and have shown that alcohol use has effects on the course of HIV, including adherence to ART. The prevalence of HIV is considerable higher in adults who have a history of alcohol consumption or frequently use alcohol than in adults who do not use alcohol [7-10]. A systematic review and meta-analysis by Fisher and colleagues confirmed the strong association between alcohol use and HIV infection [11].

The negative effects of alcohol use on the individuals are numerous. High alcohol use is associated with high-risk sexual behavior and risk of HIV [12,13]. Once individuals are infected with HIV, alcohol use is related to low participation in prevention mother-to-child transmission (PMTCT), pre-test and post-test counseling (including returning for test results) [14,15]. Overall, there is a late presentation to HIV care in patients that use alcohol, compared with those that do not use alcohol (odds ratio = 3.55, 95% confidence interval = 1.63 to 7.71) [16]. Adherence to ART is poor if alcohol is used [17-21].

HIV disease progression is accelerated by alcohol use, which is shown by a more likely decline of CD4 T cells to ≤200 cells/μl and a more frequent detectable viral load in frequent alcohol users, independent of baseline CD4 cell count and HIV viral load, ART use over time, the time since HIV diagnosis, and age and gender [22-24]. Alcohol use can cause liver problems, which could add to the risk of toxicity in patients using ART.

Screening and brief intervention for alcohol reduction in primary care patients has proven effective in reducing alcohol consumption at 6 and 12 months [25]. Results from studies on screening and brief intervention in HIV patients using alcohol, mainly performed in the USA, are not conclusive. One study performed in New York showed improvement in self-report and biological markers (CD4 cell count and HIV viral load) after an eight-session behavioral intervention after 3 months. However, this result was not sustained at 6 months [26]. A multicomponent (including behavioral) intervention showed no significant differences in adherence, CD4 count, viral load or alcohol consumption [27].

Very few reports from resource-limited settings have been published on interventions to reduce alcohol use in HIV-positive patients. One study describes an intervention based on a culturally adapted, six-session (over a 3-month period) cognitive-behavioral therapy to reduce alcohol use among HIV-infected outpatients in western Kenya, whereby the percentages of days abstinent from alcohol before session 1 were 52 to 100% for women and 21 to 36% for men. By session 6 this was 96 to 100% for women and 89 to 100% for men. The percentages of days abstinent from alcohol effect sizes (Cohen’s *d*) between the first and last cognitive-behavioral therapy sessions were 2.32 for women and 2.64 for men [28].

This study will measure the prevalence of alcohol use in patients with HIV and assess the efficacy of a brief intervention to reduce alcohol use among selected patients.

## Methods/Design

### Design

The first part of the study will be a screening phase to estimate the prevalence of (hazardous and harmful) alcohol use in the study population. The study design for the efficacy study is a single-blinded randomized controlled trial where information will be collected from patients at inclusion (before the intervention, which is performed in the same session: pre-intervention) and at follow-up of 3 and 12 months.

### Setting, study population and participants

The sample will include out-patients with HIV at the primary care clinics in townships in Gauteng, South Africa. Out-patients with HIV will be screened for hazardous and harmful alcohol use. Patients identified as hazardous drinkers (on the Alcohol Disorder Identification Test (AUDIT) questionnaire) will be randomized into an intervention group or a control group.

### Aim of the study

The aim of the study is to assess the prevalence and severity (hazardous and harmful) of alcohol use in patients with HIV and its associated factors, and to study the efficacy of a brief intervention for hazardous alcohol use in out-patients with HIV in primary health care clinics in townships in South Africa.

### Objectives

The objectives of the screening phase are: to measure the prevalence of alcohol consumption among out-patients with HIV; and to describe drinking patterns, health status, quality of life, sexual behavior and adherence levels among out-patients with HIV, and identify hazardous drinkers needing intervention.

The objectives of the intervention phase are: to compare the level of alcohol consumption, health status, quality of life, level of adherence to ART and sexual behavior among hazardous drinkers between pre-intervention and 3 and 12 months after the intervention; and to compare levels of and changes in alcohol consumption, adherence level to ART, quality of life, health status and number of patients with virological and immunological failure, between intervention and control groups at 3 and 12 months.

### Hypothesis

Hazardous drinkers with HIV in the intervention group will reduce alcohol drinking more than those in the control group. In patients that reduce alcohol use, a positive effect will be seen in adherence levels to ART, quality of life, health status and virological and immunological response.

### Inclusion criteria

Patients who are 18 years and older, with HIV-1 infection and who are visiting selected outpatient clinics for their HIV care will be eligible for this study. Patients with hazardous alcohol use will be selected using the AUDIT questionnaire (hazardous drinkers score 8 to 19 for men and 7 to 19 for women).

### Exclusion criteria

Patients with mental impairment, patients already undergoing alcohol reduction treatment and pregnant women will be excluded from this study.

### Sample size calculation

The sample size was calculated using Open Epi [29]. Based on the current AUDIT score of 12 among HIV patients, it is assumed that the intervention will reduce the current AUDIT score by 12% to 10.6 [30,31]. Based on this assumption, the estimated sample size will allow us with 80% power (5% level of significance) to detect the difference of 12% between the two groups. This will give a minimum of 99 patients per arm. It is expected that 20% of participants may be lost prior to completing the 3-month and 12-month follow-up assessments so that the final sample would be 120 per arm. A total of 240 HIV patients (with AUDIT scores 8 to 19 for men or 7 to 19 for women) will be recruited for the study.

### Randomization

Patients fulfilling criteria for selection for this study will be randomized to receive an intervention or to be in the control group. Randomization will be done by concealed, centrally allocated, computer-generated random numbers.

### Procedure

All consecutive HIV-positive patients visiting two out-patient clinics will be asked to provide informed consent to receive a baseline assessment (using the data collection instruments) and screening for alcohol problems. If eligible – meaning an AUDIT questionnaire score of 8 to 19 for men or 7 to 19 for women – a second informed consent will be asked before randomization into the intervention or control group. Patients with a score of 20 and above on the AUDIT (with probable alcohol dependence) will be referred for further management (according to local standard procedures). The research assistant nurse will randomize patients by checking the random numbers that allocate the case to the intervention arm or control arms. The intervention or control procedures will be carried out in all patients, after which they will be followed up at 3 and 12 months (which are standard visit intervals for the follow-up of the HIV care). The research assistant performing the follow-up assessments will be blinded to the intervention allocation of the participant. In the event of a drop-out, at least six individual attempts will be made to contact patients by telephone and letter. Even if a contact was not successful at either follow-up point, further attempts will be made at any next follow-up point. Sampling will occur throughout all hours of clinic operation over a 3-month period. Patients will be offered a drink (soda, juice) during the interviews.

### Data collection instruments

Eight instruments (questionnaires) will be used to collect data. All questionnaires will be administered in English or Tswana, the two languages predominantly spoken by nearly all clinic patients. The questionnaires will be administered at baseline and every follow up visit.

#### Sociodemographic characteristics

A researcher-designed questionnaire will be used to record information on participants’ age, gender, population group, language, educational level, marital status, highest educational qualification, source of income and residential status.

#### Health-related quality of life

The WHOQoL-HIVBREF is based on the WHOQOL-HIV measure, one of the two World Health Organization (WHO) quality-of-life instruments for the use in HIV-infected populations [32,33]. The WHOQoL-HIVBREF evaluates the quality of life in six domains and 31 questions.

#### Depression

Symptoms of depression will be assessed using the 10-item version of the Centers for Epidemiologic Studies Depression Scale [34]. The Centers for Epidemiologic Studies Depression Scale has been widely used in studies on the relationship between HIV and depression [35]. The sensitivity and specificity of the Centers for Epidemiologic Studies Depression Scale 20-item survey has been reported to average 80% and 70%, respectively, compared with formal diagnostic interview [36]. We will also identify patients who experience more severe depressive symptoms by distinguishing those scoring ≥15 out of 30 on the Centers for Epidemiologic Studies Depression Scale 10-item survey [34].

#### Sexual behavior

To study the sexual behavior of HIV patients, and specifically those who consume hazardous amounts of alcohol before and after intervention, we included questions on sexual behavior.

#### Internalized AIDS stigma

We will use the seven-item internalized AIDS-related stigma scale for people infected with HIV. Items reflect self-defacing beliefs and negative perceptions of people living with HIV/AIDS. In previous studies including South Africa, with the same scale, a Cronbach alpha reliability coefficient of 0.72 to 0.76 was found [37].

#### Alcohol consumption

The 10-item AUDIT assesses alcohol consumption level (three items), symptoms of alcohol dependence (three items), and problems associated with alcohol use (four items) [38]. Responses to items on the AUDIT are rated on a four-point Likert scale from 0 to 4, with a maximum score of 40 points. AUDIT scores higher than 19 indicate more severe levels of risk; scores of 8 to 19 in men and 7 to 19 in women indicate a tendency to problematic drinking. The AUDIT shows excellent sensitivity and specificity in detecting Mini-International Neuropsychiatric Interview-defined dependence/abuse (area under the receiver-operating characteristic curve = 0.96) in HIV patients in South Africa [5].

To reduce the stigma of alcohol use, the WHO suggests integrating the screening of alcohol use with screening for other health-related behaviors [38]. For this reason, two questions will be asked on the use of tobacco products and anthropometric measurements will be taken to assess the risk factor of overweight (height, weight and waist circumference).

#### HIV-related information

A researcher-designed patient information extraction sheet will be used to record information related to the HIV infection and will include questions about date of first positive HIV test, start date for ART, ART regimen, co-infections and co-medication. The patient file will be used to obtain additional information. The computerized laboratory data system will be used to obtain the laboratory results. At follow-up, CD4 cell counts and viral load will be obtained from the computerized laboratory data system.

#### Assessment of adherence to antiretroviral therapy

For assessing adherence to ART we will use three tools.

##### Self-reported adherence by recall of missed doses during last 7 days

This tool is easy to use and is significantly associated with virological and immunological outcomes [39].

##### Visual Analog Scale

The 30-day Visual Analog Scale provides an overall adherence assessment for a 1-month period. The Visual Analog Scale has been validated in resource-limited settings [40,41]. Adherence levels assessed from the Visual Analog Scale are defined as follows: full adherence, 100%; partial adherence, between ≥95% and <100%; and non-adherence, <95% of prescribed doses taken in the past 30 days. The Visual Analog Scale will be assessed at baseline for patients who are already on ART and at any follow-up visit if the patient is on ART.

##### Pill count

A pill count compares the number of pills that were dispensed and that are remaining at the pill count. A percentage of adherence to ART will be calculated.

### Interventions

#### Control group

Patients in the control group will only receive a health education leaflet on responsible drinking. They will receive the brief intervention only after 12-month follow-up, if the intervention has proven to be successful.

#### Intervention group

Patients in the intervention group will first receive personalized feedback on the AUDIT results. A health education leaflet on responsible drinking will then be given and discussed. Subsequently, simple advice and brief counseling on reducing excessive drinking will be offered during a one-session, 20-minute intervention. Since there is a serious lack of resources in South Africa in HIV care, an efficient intervention has to be short and simple to incorporate into daily practice. Intervention methods whereby patients have to come back for more sessions might in daily practice be unsuccessful because of the time-consuming aspect for those who perform the intervention and because of an expected high patient drop-out rate. A short, one-session intervention has therefore been chosen to be tested in this trial. The brief intervention is based on the WHO brief intervention package for hazardous and harmful drinking [42], which is based on the Information–Motivation–Behavioural Skills Model. The steps of brief counseling are: identify any alcohol-related problems mentioned in the interview; introduce the sensible drinking leaflet, and emphasize the idea of sensible limits, and make sure that patients realize that they are in the medium-risk drinking category; work through the first three sections of the problem-solving manual while mentioning the value of reviewing the other sections; describe drinking diary cards; identify a helper; and mention the 3-month and 12-month follow-up.

### Counselor training and intervention quality assurance

The intervention research assistants will be trained to deliver the interventions to the patients. The training consists of role play, general skills training techniques and training on HIV and sexual-related issues. A comprehensive manual for the brief intervention is available and will be used to guide the counseling session. The sites will be visited twice a week by one of the investigators to observe whether there is adherence to the protocol and to offer support and supervision to the research assistants. During regular research meetings, occurring problems will be discussed and solved.

### Data analysis

Virological failure will be defined according to the definition in the South African national guidelines on HIV: a repeat HIV viral load ≥1,000 copies/ml after intense adherence assessment [43]. Immunological failure will be defined according to the WHO definition: a fall of CD4 cell count to pre-therapy baseline (or below), or a 50% fall from the on-treatment peak value (if known), or a persistent CD4 cell count level below 100 cells/mm^3^[44].

Means, standard deviations, and percentages will be used for descriptive statistics. To examine differences between groups, the *t* test for continuous data and the chi-square test for categorical data will be used. Generalized linear model repeated measures 2×3 analysis of variance will be used for comparing observations (alcohol use score) across the three contact periods to demonstrate a treatment intervention×time interaction. To avoid possible bias from excluding patients with missing values, patterns of missing values will be analyzed. Multiple imputation methods using generalized estimating equating methods, based on all data available in the model, using five imputed datasets, will be used to impute missing data at all contact points (baseline and follow-up at 3 and 12 months). SPSS for Windows version 18.0 (SPSS, Inc., Chicago, IL, USA) will be used for calculations.

### Outcomes

The findings will be important in the public health setting. The AUDIT screening tool has been developed by the WHO and proved to be applicable in a wide range of settings and target groups, including HIV patients in South Africa. The brief intervention we propose was also developed by the WHO and was proven to be successful in different settings [42]. This makes the intervention widely applicable in different settings and regions. However, the intervention was never tested among patients with HIV infection in South Africa, which this trial aims to do. If the intervention proves to be efficient, it could potentially be incorporated in the South African HIV care guidelines. The generalizability of the trial result may not be extended to other countries, however, where there might be different patterns of alcohol consumption and different cultural beliefs regarding alcohol use and medical care.

### Trial status

Patients are currently being recruited for the trial.

### Ethical and governance approval

Ethical approval was given by the Medunsa Research and Ethics Committee (Project number: MREC/H/165/2011: IR). The Tshwane Research Committee has provided approval for the study (Project number: 2011/46).

## Abbreviations

AIDS: acquired immune deficiency syndrome; ART: antiretroviral therapy; AUDIT: Alcohol Use Disorder Identification Test; HIV: human immunodeficiency syndrome; WHO: World Health Organization.

## Competing interests

The authors declare that they have no competing interests.

## Authors’ contributions

All authors contributed to the design of the study. DHitV, KP and SP contributed to the training of the research assistants. DHitV and LS contributed to the quality control and supervision of research assistants. DHitV contributed to the data collection, statistical analysis and drafting of the manuscript. All authors read, commented on and approved the final manuscript.
